# Sternal wound infection caused by *Gordonia bronchialis*: identification by MALDI-TOF MS

**DOI:** 10.1099/jmmcr.0.005067

**Published:** 2016-10-31

**Authors:** Jesús Rodriguez-Lozano, Enrique Pérez-Llantada, Jesús Agüero, Ana Rodríguez-Fernández, Carlos Ruiz de Alegria, Luis Martinez-Martinez, Jorge Calvo

**Affiliations:** Marqués de Valdecilla University Hospital, Santander, Cantabria, Spain

**Keywords:** *Gordonia*, MALDI-TOF, sternal wound infection

## Abstract

**Introduction:**

*Gordonia* spp. infections are uncommon. However, a few clinical cases have been reported in the literature, particularly those involving immunocompromised hosts. Advanced microbiology diagnosis techniques, such as matrix-assisted laser desorption ionization-time of flight MS (MALDI-TOF MS), have been recently introduced in clinical microbiology laboratories in order to improve microbial identification, resulting in better patient management.

**Case presentation:**

Here, we present a new clinical case of persistent wound infection caused by *Gordonia bronchialis* in a 64-year-old woman after a mitral valve replacement, using two MALDI-TOF-based systems for identifying this micro-organism.

**Conclusion:**

Both MALDI-TOF systems were able to identify *Gordonia* spp.; thus, providing a useful tool that overcomes the current limitations of phenotypic identification associated with this micro-organism. Although the technique validation deserves additional verification, our study provides guidance about MALDI-TOF as a fast and easy method for *Gordonia* spp. identification.

## Introduction

*Gordonia* species are aerobic actinomycetes that rarely infect humans, incidences that do occur are most notably in the setting of intravascular catheter-related infections (Johnson *et al.*, 2011) and usually involve immunocompromised patients. There are approximately 35 validly named species in the genus (Conville & Witebsky, 2015), and the most frequently isolated species from human infections are *Gordonia aichiensis*, *G. araii*, *G. bronchialis*, *G. otitidis*, *G. polyisoprenivorans*, *G. rubripertincta*, *G. sputi* and *G. terrae* (Guerrero Gómez *et al.*, 2014). *G. bronchialis* was first identified in soil samples, as well as in sputum samples obtained from patients with pulmonary disease (Tsukamura, 1971).

We present a case of persistent sternal wound infection by *G. bronchialis* in an immunocompetent woman. Our main purpose is to show our experience and published ones in applying matrix-assisted laser desorption ionization-time of flight MS (MALDI-TOF MS) technology to identify this micro-organism.

## Case report

A 64-year-old woman was admitted to hospital because of dehiscence of a sternal wound, after a mitral valve replacement that was performed 2 months earlier due to severe insufficiency. She presented a clinical history of rheumatic mitral stenosis, which was treated with closed mitral valvulotomy 35 years previously, resulting in a mitral insufficiency. Twenty-three years previously she had suffered a bacterial endocarditis due to viridans group streptococci that led to cerebral embolism.

On examination, a white material was found to be exuded from the sternal wound when pressed over the wound margins. A computed tomography scan of the chest showed a dehiscence of the surgical wound, with swelling of soft tissue above the sternum and osteitis of the sternal bone. Apart from a C-reactive protein level of 2.6 mg dl^−1^ and an albumin level of 3.1 g dl^−1^, laboratory studies were unremarkable.

Empirical treatment with clindamycin (300 mg/6h i.v.) and ceftazidime (2 g/8h i.v.) was started. The treatment was changed to imipenem (500 mg/6h i.v.) and ciprofloxacin (750 mg/12h p.o.) after a preliminary microbiology laboratory report of growth of an actinomycete with presumed susceptibility to several antimicrobials. Surgical debridement of the wound was performed. This treatment was maintained for 3 weeks, but successive wound cultures continued showing the presence of the actinomycete organism. Because the symptoms did not improve, sternal cerclage was removed and antibiotic therapy was shifted to teicoplanin (400 mg/24h i.v.) plus ciprofloxacin (750 mg/12h p.o.) and rifampin (600 mg/24h p.o.) for 2 weeks, followed by ciprofloxacin plus rifampin for an additional6 weeks, resulting in wound healing.

**Fig. 1.**
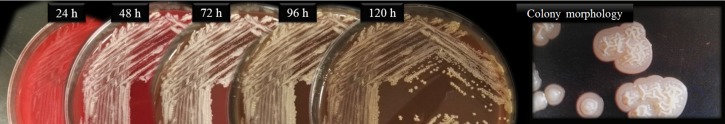
Evolution of colony appearance.

Culture of wound samples on chocolate and blood agar plates for 72 h at 37 °C in aerobic conditions yielded creamy-white, dry, wrinkled and non-haemolytic colonies. After these 3 days, a colour change was observed in the colonies from white to yellowish. Colony appearance showed synnemata and no aerial hyphae (see Fig. 1). Gram staining yielded Gram-positive short coryneform rods without branching. Modified Ziehl–Neelsen staining confirmed slight acid-fastness. Both conventional Ziehl–Neelsen and auramine stains were negative. The micro-organism was non-spore-forming, and catalase and urease positive. Casein, hypoxanthine, tyrosine and gelatine were not decomposed. Arylsulfatase production was negative within 3 days. Nitrate was not reduced to nitrite and indole was not produced. With the API NH strip (bioMérieux) acid was produced from glucose, fructose and sucrose. 16S rRNA gene sequence analysis using the blast algorithm showed 99.9 % similarity to *G. bronchialis* strain DSM 43247 (GenBank accession no. NR074529.1).

An antimicrobial-susceptibility assay was performed using Etest strips (bioMérieux) on Mueller–Hinton agar with 5 % defibrinated horse blood and 20 mg β-NAD l^−1^ (MH-F; Oxoid). Readings were taken after 48 h of incubation, and susceptibility categories were defined according to Clinical and Laboratory Standards Institute (CLSI) guidelines for mycobacteria, nocardiae and other actinomycetes (CLSI, 2011). The isolate was resistant to clindamycin (MIC=8 mg l^−1^), and susceptible to amoxicillin/clavulanic (0.016 mg l^−1^), ceftriaxone (0.5 mg l^−1^), imipenem (0.008 mg l^−1^), ciprofloxacin (0.06 mg l^−1^), amikacin (0.06 mg l^−1^), tobramycin (0.12 mg l^−1^), clarithromycin (2 mg l^−1^), minocycline (0.25 mg l^−1^), linezolid (1 mg l^−1^) and co-trimoxazole (0.03 mg l^−1^). Although no susceptibility breakpoints have been established for vancomycin and teicoplanin by the CLSI, MIC values were low (0.25 and 1 mg l^−1^, respectively).

The isolate was analysed by two MALDI-TOF MS-based systems, a Bruker Biotyper (Bruker Daltonics) and a Vitek MS (bioMérieux). Identification of *G. bronchialis* (99.9 % identity) was obtained with the Vitek MS (saramis 3.0 software) following the procedure recommended by the manufacturer. Briefly, target slides were inoculated into the spots by picking a freshly grown overnight colony and overlaid with 1 µl matrix solution, α-cyano-4-hydroxycinnamic acid. The same result was attained with the Bruker Biotyper (version 3.1 software), using a complete protocol of protein extraction with formic acid and acetonitrile, following the Bruker Biotyper instructions, but the score value (1.72) was lower than the one defined in the manufacturer’s criteria (≥2.00) for acceptance of identification at the species level.

## Discussion

*Gordonia* is a Gram-positive rod with mycolic acids in its structure, which confer partially acid-fast staining. It has been reported to cause a variety of infections after coronary bypass surgery, such as sternal wound infection, bacteraemia, osteomyelitis, pleural infection and recurrent breast abscess (Richet *et al.*, 1991; Sng *et al.*, 2004; Werno *et al.*, 2005; Johnson *et al.*, 2011; Siddiqui *et al.*, 2012; Guerrero Gómez *et al.*, 2014).

Other cases of sternal wound infection by *G. bronchialis* have been reported previously and are summarized in Table 1. All cases had a history of previous cardiac surgery, including two outbreaks probably related to intraoperative transmission from a nurse.

**Table 1.** Summary of reported cases of sternal wound infection due to *G. bronchialis*

Case	No. of patients	Age (years)	Sex	Underlying condition or risk	Clinical manifestation	Treatment (duration)	Identification method	Reference
1	7 (cluster)	51–68	Male	Coronary-artery bypass surgery	Blister or a localized area of inflammation; purulent drainage of the sternal wound	Patient 1, ciprofloxacin p.o. (74 days); patient 2, co-trimoxazole p.o. (122 days); patient 3, ceftriaxone i.v. (38 days) and ciprofloxacin p.o. (108 days); rest of patients required surgical debridement and oral antimicrobial therapy	Conventional biochemical test	Richet *et al.* (1991)
2	3 (cluster)	56–80	Male	Coronary-artery bypass surgery	Deep sternal infection	Imipenem i.v. (41–77 days); one patient received additional oral antibiotics – moxifloxacin, linezolid and minocycline (56 days); wound debridement and flap grafts	16S rRNA sequencing	Wright *et al.* (2012)
3	1	76	Female	Coronary-artery bypass surgery	External fistulation and inflammatory signs of surgical wound; osteomyelitis	Ceftriaxone i.v. (21 days); ciprofloxacin p.o. (14 days); Wound debridement and V.A.C therapy.	16S rRNA sequencing	Vasquez *et al.* (2013)
4	1	69	Female	Coronary-artery bypass surgery	Sternotomy site pain; redness, tenderness and pus on the operation site	Empirical treatment – vancomycin and cefotetan (duration na); treatment after preliminary results – penicillin (duration na); final treatment – imipenem (56 days)	16S rRNA sequencing	Chang *et al.* (2014)
5	4 (3 *G. bronchialis* and 1 *G. terrae)*	47–68	Male/ female	Coronary-artery bypass surgery	Drainage from the incision; pain, redness or swelling at the sternal wound	Wound debridement and negative-pressure wound therapy; antimicrobial therapy not described	na	Nguyen *et al.* (2014)
6	1	64	Female	Mitral valve replacement	Dehiscence and white material from the sternal wound	Empirical treatment – clindamycin and ceftazidime; treatment after preliminary results – imipenem i.v. and ciprofloxacin p.o.; final treatment– teicoplanin, ciprofloxacin and rifampin (14 days), followed by ciprofloxacin plus rifampin (42 days)	16S rRNA sequencing	Present study (2016)

i.v., Intravenous; na, not available; p.o., oral.

Phenotypic identification of *Gordonia* spp. is not conclusive, and biochemical profiles can lead to incorrect identification of isolates as non-tuberculosis mycobacteria or other actinomycetes, especially with the genus Rhodococcus, since both are coryneform, aerial-hyphae negative and weak modified acid-fast. Molecular methods, such as 16S rRNA gene sequencing, have significantly improved organism identification, but the results are not generated in a short time and these methods are not available in all laboratories. Recently, MS-based systems emerged as a reliable, fast and cost-effective tool for the identification of bacteria and fungi in routine diagnosis.

Few studies have evaluated the accuracy of MALDI-TOF MS for the identification of *Gordonia*. Vasquez *et al.* (2013) reported the first clinical diagnostic identification of *G. bronchialis* by applying a Bruker Biotyper (score values of 1.515 and 1.892 using different methods of sample preparation). Hsueh *et al.* (2014) evaluated the performance of the Bruker Biotyper with seven strains of *Gordonia* species. The system identified two out of three *G. bronchialis* and a *G. sputi* isolate, but the identification scores obtained were below 2. Moreover, three *Gordonia amicalis* were identified as *G. rubripertincta,* with score values lower than 1.7, because *G. amicalis* is not included in the Bruker Biotyper 3.1 software. Lam *et al.* (2015) reported the reliability of the Bruker Biotyper in identifying two *G. sputi* strains (scores 2.039 and 2.026) and one *G. bronchialis* (score 1.743). Both research groups used a complete protocol of extraction with formic acid and acetonitrile following the Bruker Biotyper instructions, as in our case.

Other authors, such as Barberis *et al.* (2014), applied a simplified method of extraction for the Bruker Biotyper system, as previously described (a colony was inoculated on the MALDI-TOF plate and sequentially overlaid with 0.5 µl formic acid and 1 µl matrix; Theel *et al.*, 2012), to a collection of Gram-positive rods of clinical origin, including three *Gordonia* strains (none were *G. bronchialis*). Only one *G. terrae* strain was identified with a score ≥2.0, but following published recommendations (Alatoom *et al.*, 2012; Bizzini *et al.,* 2011), an additional identification of *G. terrae* was accepted at the species level. These recommendations apply lower cut-off scores for identification of Gram-positive rods (≥1.5 for the genus level and ≥1.7 for the species level). We repeatedly tried to identify our isolate by this simplified extraction method, but no result was obtained in any case. Titécat *et al.* (2014) failed to identify *G. bronchialis* with the Bruker Biotyper system with an easier sample preparation method (score lower than 1.5). They applied a small amount of bacterial sample on the target plate, which was overlaid only with 1 µl matrix without adding formic acid. Retesting the isolate with the recommended protein extraction method yielded an identification of *Arthrobacter castelii* with a score of 1.967.

A PubMed search using *Gordonia* and Vitek MS as keywords yielded no published studies evaluating the performance of this system for *Gordonia* identification. However, our clinical isolate was identified by Vitek MS as *G. bronchialis* with a high level of identity, applying an easier method of sample preparation compared to the one recommended by the manufacturer.

In summary, although more information is required about the reliability of MALDI-TOF MS for the identification of *Gordonia* species, and other actinomycetes in general, it would be expected that more cases of infections by these micro-organisms could be diagnosed with these new identification approaches. In this aim, a high degree of suspicion for the presence of these micro-organisms in the sample, after direct staining, is important in order to prolong the incubation of cultures.
